# Winkelstabile Plattenosteosynthese bei distalen periprothetischen Femurfrakturen: Klinisches Outcome und Mortalität

**DOI:** 10.1007/s00113-020-00911-6

**Published:** 2020-11-20

**Authors:** C. Gassner, F. Sommer, B. Rubenbauer, A. M. Keppler, Y. Liesaus, W. C. Prall, C. Kammerlander, W. Böcker, J. Fürmetz

**Affiliations:** 1 Klinik für Allgemeine, Unfall- und Wiederherstellungschirurgie, Klinikum der Ludwig-Maximilians-Universität München, Marchioninistr. 15, 81377 München, Deutschland; 2 Zentrum für Knie‑, Hüft- und Schulterchirurgie, Schön Klinik München Harlaching, Harlachinger Str. 51, 81547 München, Deutschland

**Keywords:** Alterstraumatologie, Knieprothese, Rorabeck-Klassifikation, Osteosynthese vs. distaler Femurersatz, Mortalität, Geriatric traumatology, Knee prosthesis, Rorabeck classification, Osteosynthesis vs. distal femoral replacement, Mortality

## Abstract

**Hintergrund:**

Die Versorgung distaler periprothetischer Femurfrakturen (PFF) stellt aufgrund des geriatrischen Patientenkollektivs eine große interdisziplinäre Herausforderung dar und erfolgt (abhängig vom Frakturtyp) häufig mittels winkelstabiler Plattenosteosynthese (WPO), wobei bisher nur wenige Daten zum klinischen Outcome existieren. Ziel der Untersuchung ist die Identifikation von Risikofaktoren für ein schlechtes Outcome und erhöhte Mortalität.

**Methoden:**

In dieser retrospektiven Studie wurden 36 Fälle mit distaler PFF untersucht. Eingeschlossen wurden nur Versorgungen mit WPO. Neben relevanten Vorerkrankungen (ASA-Score, Charlson Index), der Frakturmorphologie und relevante Komplikationen, wurden u.a. die 1- und 3-Jahres-Mortalität, sowie das klinische Outcome mit Hilfe des Lysholm-Scores erfasst.

**Ergebnisse:**

Die 1- und 3- Jahres Mortalität betrug 9% bzw. 26%, wobei dies ausschließlich ASA 3 und 4 Patienten betraf. Der Lysholm Score zeigte eine hohe Variabilität (65 ± 27 Punkte) mit höheren Werten in der ASA 1-2 Subgruppe (82 vs. 63 Punkte) aber unabhängig vom Frakturtyp. Als Risikofaktoren für die 3-Jahres Mortalität konnten der präoperative ASA-Score, der Charlson Komorbiditätsindex und das Patientenalter identifiziert werden.

**Diskussion:**

Die dargestellte Fallserie weist eine hohe absolute Mortalitätsrate auf, auch wenn diese im Vergleich zu bisher publizierten Daten, etwas geringer war. Die Rate an Sekundärdislokationen, fehlender Frakturheilung oder Folgeoperationen war ebenfalls gering. Die WPO erscheint daher als geeignete Versorgung für Frakturen mit stabiler Prothese. Jedoch besteht eine hohe Variabilität im klinischen Outcome unabhängig vom Frakturtyp, sowie signifikant erhöhte Mortalitätsraten bei vorerkrankten Patienten.

## Abstract (en)

**Background:**

The care of distal periprosthetic femoral fractures (PFF) is becoming a major interdisciplinary challenge due to demographic developments. The operative treatment is often performed (depending on the type of fracture) by means of locking plate fixation (LPF), although little data on the clinical outcome exist by now. The aim of the study is to identify risk factors for a poor outcome and increased mortality

**Methods:**

In this retrospective study, 36 cases with distal PFF were examined. Exclusively treatment with LPF were included. Relevant previous illnesses (ASA score, Charlson index), fracture morphology and major complications were recorded as well as 1- and 3- year mortality. The clinical outcome was detected by using the Lysholm score.

**Results:**

The 1- and 3- year mortality were 9% and 26% - exclusively affecting ASA 3 and 4 patients. The Lysholm Score showed a high variability (65 ± 27 points) with higher values in the ASA 1-2 subgroup (82 vs. 63 points) but independent of fracture type. The preoperative ASA score, the Charlson comorbidity index, and the patient age were determined to be decisive for 3-year mortality.

**Conclusion:**

This case series displayed a high absolute mortality even if the rate was slightly lower compared to previously published data. The rate of secondary dislocations, lack of fracture healing or follow-up operations were also low. The LPF therefore appears to be a suitable treatment for fractures with a stable prosthesis. However, there is a high variability in the clinical outcome regardless of the type of fracture and significantly increased mortality rates in previously ill patients.

## Einleitung

Im Jahr 2016 wurden in Deutschland 233.424 Hüftprothesen und 187.319 Knieprothesen implantiert [13]. Sie gehören mit jährlich steigender Tendenz zu den häufigsten Eingriffen (Platz 6 bzw. 14) in Deutschland. Aufgrund der immer höheren Lebenserwartung und des steigenden Aktivitätsniveaus im Alter ist mit einem weiteren Anstieg der Implantationen zu rechnen [9]. Verbunden damit werden auch Komplikationen wie Infektionen/Wundheilungsstörungen, Prothesenlockerungen, Luxationen und periprothetische Frakturen zunehmen [26]. Vor allem die prothesennahen Frakturen stellen eine große Herausforderung in der Orthopädie und Unfallchirurgie dar. Im Hüftbereich wird die Rate an periprothetischen Frakturen mit 1,1 % nach Primär- und 4,0 % nach Revisionseingriffen beziffert, während das Risiko am Kniegelenk mit 2,2 % bzw. 4,4 % etwas höher angegeben wird [2]. Hierbei stehen v. a. Frakturen am distalen Femur im Vordergrund mit einer ca. 10-mal höheren Wahrscheinlichkeit gegenüber Patella- und Tibiakopffrakturen [3]. Die häufigste Ursache sind niedrig-energetische Traumamechanismen bei bestehender Osteoporose [1, 10].

Das durchschnittliche Alter von Patienten mit einer distalen periprothetischen Femurfraktur liegt bei 76 Jahren, im Gegensatz zu 80 Jahren am proximalen Femur [6]. Aufgrund des hohen Patientenalters bestehen neben der Osteoporose oft zahlreiche weitere Komorbiditäten. Weitere Risikofaktoren, eine periprothetische Femurfraktur zu erleiden, sind bereits stattgehabte operative Revisionen, Infektionen, Achsfehler und Malalignment der Prothesenkomponenten, chronische Prothesenlockerung sowie internistische und neurologische Begleitumstände, die das Sturzrisiko von Patienten erhöhen [26]. Begleiterkrankungen begünstigen jedoch nicht nur das Auftreten von Frakturen, sondern erhöhen ebenfalls das intra- und postoperative Risiko [32]. Häufig sind die Patienten bereits vor dem Sturz in ihrer Mobilität eingeschränkt und können deswegen postoperativ deutlich schwerer mobilisiert werden. Alle diese Faktoren führen insgesamt zu einem hohen Mortalitätsrisiko in dieser Patientengruppe [15, 29].

**Figure 1:**
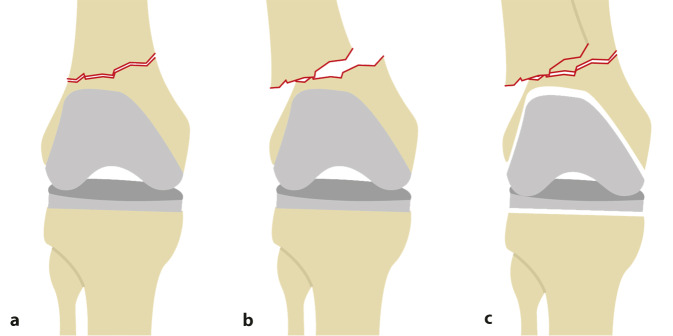

Mehrere Klassifikationen zur Einteilung der distalen periprothetischen Femurfrakturen wurden beschrieben, von denen die etabliertesten auf Rorabeck und Taylor sowie auf Su zurückgehen (Abb. 1; [23, 24, 30]). Eine entscheidende Rolle für die operative Versorgungsstrategie spielt dabei die Frakturmorphologie. Frakturen proximal des Prothesenschildes eignen sich auch für eine Nagelosteosynthese, falls das Prothesendesign dies zulässt [30]. Wird dieses Verfahren gewählt, steht eine Lockerung der Prothese meist nicht zur Diskussion. Sobald die Fraktur weiter nach distal reicht, bietet die winkelstabile Plattenosteosynthese gerade bei geringer verbleibender Knochensubstanz mehr Fixierungsmöglichkeiten, und es werden ähnliche Ergebnisse wie bei den weiter proximal gelegenen Frakturen erreicht [28]. Eine Prothesenlockerung der femoralen Prothesenkomponente sollte einen Prothesenwechsel nach sich ziehen und bedarf dann eines anderen Zugangs und der Vorhaltung von Implantaten [9, 20, 24]. Deshalb sollte die Lockerung präoperativ anhand der CT-Bilder erkannt werden, was in der Praxis nicht immer sicher möglich ist. Eine konservative Therapie ist aufgrund eingeschränkter Mobilisierungsmöglichkeit und einer hohen Pseudarthrosenrate nur bei infauster, palliativer Situation indiziert [9].

In der vorliegenden Fallserie werden Patienten mit distalen periprothetischen Femurfrakturen und Versorgung mit winkelstabiler Plattenosteosynthese retrospektiv nachuntersucht. Es sollen sowohl Risikofaktoren für eine erhöhte Mortalität identifiziert als auch Daten über das klinische Outcome erhoben werden.

## Material und Methoden

In dieser retrospektiven Fallserie wurden Patienten mit distaler periprothetischer Femurfraktur nachuntersucht, die mittels winkelstabiler Plattenosteosynthese im Zeitraum 2010–2016 operativ versorgt wurden. Das minimale Follow-up nach der Operation betrug 3 Jahre. Ein positives Votum der lokalen Ethikkommission lag vor Beginn der Untersuchung vor (EK-Nr: 19-241). Für den Auswertungszeitraum wurden über die Klinikumdatenbank Patienten identifiziert, die mit der ICD-10 distale Femurfraktur (S72.4) stationär behandelt wurden. Darunter wurden nur die periprothetischen Frakturen isoliert. Von diesen Patienten wurden die initialen Röntgen- und CT-Untersuchungen sowie die Arztbriefe der stationären Behandlung und die Operationsberichte ausgewertet. Einschlusskriterium war eine radiologisch nachgewiesene und operativ mittels winkelstabiler Platte (LISS-Platte: Fa. DePuy Synthes, Raynham, MA, USA; NCB-Platte: Fa. Zimmer Biomet, Warsaw, IN, USA) versorgte distale periprothetische Femurfraktur. Ausgeschlossen wurden Fälle mit intramedullärer Stabilisierung, Knieprothesenwechsel, periprothetischer Refraktur und offene Frakturen. Die Nachbehandlung erfolgte bei allen Patienten mit der Vorgabe einer Teilbelastung von maximal 20 kg.

Allgemeine Patientencharakteristika wie Alter, Geschlecht, ASA-Klassifikation, Begleitverletzungen und BMI wurden aufgenommen (Tab. 1; [18, 27]). Zur weiteren Charakterisierung der Grundmorbidität unseren Patientenkollektivs zogen wir den Charlson-Komorbiditätsindex, der 19 Vorerkrankungen berücksichtigt, heran. Als Frakturklassifikation wurde die Einteilung von Rorabeck und Taylor (retrospektiv) verwendet [24]. Zudem wurde die Art der liegenden Knieprothese miterfasst. Zusätzlich konnte die Anzahl der Frakturfragmente in der präoperativen Computertomographie erfasst werden. Um die intraoperative Invasivität einschätzen zu können, wurden Operationsdauer und Blutverlust erhoben.

**Table 1:** 

*Gesamtkollektiv*	35 Patienten bzw. 36 Fälle
Weiblich	31
Männlich	4 Patienten bzw. 5 Fälle
*Alter [Jahre]*	81,7 (±7,0)
*ASA-Score*	2,81 (±0,58)
*BMI [kg/m*^*2*^*]*	25,2 (±4,3)
*Vorbestehende Implantate/Prothesen im ipsilat. Femur*	9‑mal Hüftprothese
	3‑mal prox. Femurnagel
*Anzahl der Knochenfragmente*	3,2 (±1,4)
*Gleichzeitig erlittene Verletzungen*	1‑mal Dens-Fraktur
	1‑mal distale Tibiafraktur
	2‑mal Unterschenkelfraktur
	1‑mal Unterschenkel- und Ulnafraktur
*Verstorben zum Zeitpunkt der Datenerhebung nach einem durchschnittlichen Nachbeobachtungszeitraum von 46,6 Monaten*	14 von 33 Patienten/34 Fälle (2 Patienten „lost to follow up“)
*Mortalität nach 3 Monaten*	2 von 33 (6 %)
*Mortalität nach 1 Jahr*	3 von 33 (9 %)
*Mortalität nach 3 Jahren*	9 von 33 (27 %)

Weitere eingeschlossene Parameter waren Besonderheiten während des Behandlungsverlaufes, wie klinisch relevante Komplikationen mit notwendiger Folgeoperation. Das radiologische Outcome wurde anhand von Röntgenverlaufskontrollen am 1. postoperativen Tag sowie 6 Wochen postoperativ und anschließend je nach Befund vorgenommen. Als klinischer Outcome-Parameter wurde der modifizierte Lysholm-Score erfasst, da dieser im älteren Patientenkollektiv einfach zu erheben ist und häufig bei kniegelenknahen Verletzungen als Verlaufsparameter dient [19, 31]. Zur Auswertung des Lysholm-Scores wurden die in der Literatur vorgegebenen Grenzwerte übernommen: 95 bis 100 Punkte exzellent, 84 bis 94 Punkte gut, 65 bis 83 Punkte befriedigend, 64 und weniger Punkte schwach [4]. Neben der Gesamtmortalität im Untersuchungszeitraum wurde auch die Mortalität innerhalb der ersten 3 Monate, des ersten Jahres und der ersten 3 Jahre erhoben. Die beschriebenen Daten konnten aus den vorhanden Patientendaten, durch klinische Nachuntersuchungen und einen Abgleich des Patientenkollektivs mit dem Sterberegister erfasst werden. Zur Erhebung des Lysholm-Scores mussten aufgrund der sehr geringen Wiedervorstellungsrate Telefoninterviews eingesetzt werden. Eine Subgruppenanalyse „Frakturklassifikation“ (Rorabeck) und „Vorerkrankungen“ (ASA) wurde hinsichtlich des klinischen Outcome, der Revisionsraten und der Mortalität durchgeführt.

Mithilfe tabellarischer Aufstellung und Grafiken erfolgte eine deskriptive Darstellung der Daten mittels Excel. SPSS 16.0 (SPSS Inc., Chicago, IL) wurde für statistische Berechnungen verwendet. Zur Berechnung des Signifikanzniveaus wurde der Mann-Whitney-U-Test verwendet (Signifikanz *p* < 0,05; KI Intervall 0,95). Metrisch skalierte Daten werden als arithmetisches Mittel mit Standardabweichung angegeben.

## Ergebnisse

### Allgemeine Charakteristika des Patientenkollektivs

Von 421 identifizierten Fällen mit distaler Femurfraktur bei sowohl nativen als auch prothetisch-versorgten Kniegelenken aus oben genanntem Zeitraum konnten letztlich 36 Fälle bei 35 Patienten eingeschlossen werden. Ein Patient erlitt im Abstand von 4 Monaten beidseitige distale periprothetische Femurfrakturen. Zum Zeitpunkt der Datenerhebung waren 14 von 33 Patienten bereits verstorben, 2 Patienten „lost to follow up“.

Das untersuchte Patientenkollektiv ist vorwiegend weiblich (31 von 35 Patienten) und weist ein Durchschnittsalter von 81,7 Jahren auf. Der durchschnittliche präoperative ASA-Score liegt bei 2,81 und beschreibt damit ein deutlich vorerkranktes Patientenkollektiv. Weitere demografische Daten sind in Tab. 1 aufgelistet.

Relevante Operationsparameter sind in Tab. 2 zusammengefasst.

**Table 2:** 

	Op. innerhalb von 48 h	Intraop. Blutverlust [ml]	Schnitt-NahtZeit [min]	Gesamte stat. Aufenthaltsdauer [Tage]
*WPO gesamt (n* *=* *36)*	23	405 (SD 252)	141 (SD 50)	16,1 (SD 8,0)

### Frakturklassifikation

In 66 % der Fälle lag eine komplexe Fraktursituation mit 3 bis 8 Knochenfragmenten vor. 29 Fälle konnten retrospektiv klar einem Rorabeck-Typ 1 oder 2 zugeordnet werden. In 7 Fällen war eine Lockerung der Prothese im konventionellen Röntgen sowie in den ergänzenden CT-Aufnahmen nicht sicher auszuschließen, weshalb diese Fälle als Typen 2 und 3 klassifiziert wurden. Intraoperative Daten bezüglich einer sicheren Lockerung liegen leider nicht vor. Die Subgruppenanalyse Frakturklassifikation nach Rorabeck zeigte keine relevanten Korrelationen hinsichtlich klinischem Outcome, Komplikationsrate und Mortalität (Tab. 3). Bis auf 3 Prothesen waren alle liegenden Knieprothesen ein Oberflächenersatz ohne Koppelung, bei 3 Patienten lagen sc-Revisionsprothesen vor.

**Table 3:** 

	Verteilung	LysholmScore	Einjahresmortalität	Dreijahresmortalität	Komplikationen
*RorabeckTyp 1*	5 (13,9 %)	64 Punkte (*n* = 4)	0 % (0 von 5)	40 % (2 von 5)	1 Epifasziale Wundheilungsstörung
*RorabeckTyp 2*	24 (66,7 %)	65 Punkte (*n* = 14)	12,5 % (3 von 24)	16,7 % (4 von 24)	2 Pseudarthrosen, 1 Infektpseudarthrose, 1 Refraktur mit Plattenbruch
*RorabeckTyp 2–3*	7 (19,4 %)	70 Punkte (*n* = 2)	0 % (0 von 5; 2 Lost to follow up)	60 % (3 von 5; 2 Lost to follow up)	1 Arthrofibrose

### Komplikationen

Unter den 36 versorgten Frakturen konnten in 24 Fällen die Daten zu Komplikationen im Verlauf bis zur Nachuntersuchung erhoben werden: Es konnten 6 Komplikationen mit notwendiger operativer Revision festgestellt werden: 2 Pseudarthrosen mit ausbleibender Frakturheilung nach 6 Monaten, eine Infektpseudarthrose, eine Refraktur mit Plattenbruch (externe Versorgung), eine Arthrofibrose und eine epifasziale Wundheilungsstörung, welche zwar operativ, aber ohne Reosteosynthese ausbehandelt werden konnte. Die Informationen über die operativ versorgten Pseudarthrosen mussten teilweise über Angehörige erfragt werden, sodass eine detaillierte Aufstellung zur Versorgung der Pseudarthrose nicht möglich ist. Risikofaktoren für eine Komplikation wie z. B. die Frakturform oder Vorerkrankungen konnten in diesem Kollektiv nicht identifiziert werden.

### Mortalitätsraten und klinisches Outcome

Die Mortalitätsraten nach 3 Monaten, einem Jahr und 3 Jahren sind in Tab. 3 aufgeführt. Als relevanter Einflussfaktur für die Mortalität wurden die Komorbiditäten identifiziert. Zudem steigt mit dem Patientenalter die Dreijahresmortalität signifikant an (*p* < 0,045). Andere mögliche Einflussfrakturen wie der intraoperative Blutverlust, die Dauer des stationären Aufenthalts und die präoperative Liegedauer zeigten keinen statistisch signifikanten Einfluss auf die Mortalität.

Trotz des hohen Patientenalters konnten wir von insgesamt 20 Patienten den Lysholm-Score erfassen, mit einem mittleren Nachuntersuchungszeitraum von 46,6 Monaten. Es zeigte sich insgesamt eine hohe Variabilität mit einer Standardabweichung von 27 Punkten bei einem durchschnittlichen Lysholm-Score von 65 Punkten.

### Komorbiditäten

Das klinische Outcome, aufgeteilt nach ASA-Score, veranschaulicht Tab. 4. Es wurde eine Subgruppenanalyse der ASA 1–2 und der ASA 3–4 durchgeführt. Hier zeigte sich ein statistisch signifikanter Gruppenunterschied in der Ein- (*p* < 0,05) und Dreijahresmortalität (*p* < 0,01).

**Table 4:** 

	Alter [Jahre]	Zeit vom Unfall bis zur Operation [Tage]	Lysholm Score (soweit vorhanden)	Einjahresmortalität [%]	Dreijahresmortalität [%]	Intraop. Blutverlust [ml]	Anteil der Op.-Dauer >2 h [%]	Stat. Aufenthalt [Tage]
*ASA-Score 1–2 (n* *=* *7)*	77	2,9	82	0	0	325	28,6	13,1
*ASA-Score 3–4 (n* *=* *26)*	82,5	2,8	63	11	34	446	62,5	16

Als weiteres Maß zu Quantifizierung und Beurteilung der Begleiterkrankungen zogen wir den Charlson-Komorbiditätsindex heran. Im Gesamtkollektiv aller Fälle zeigte sich ein Durchschnittswert von 2,2 Punkten, wobei 16 Patienten bereits 3 oder 4 Punkte aufwiesen. Auch in Bezug auf den Charlson-Komorbiditätsindex zeigte sich ein statistisch signifikanter Zusammenhang zu Ein- (*p* < 0,05) und Dreijahresmortalität (*p* < 0,01).

### Fallbeispiel

**Figure 2:**
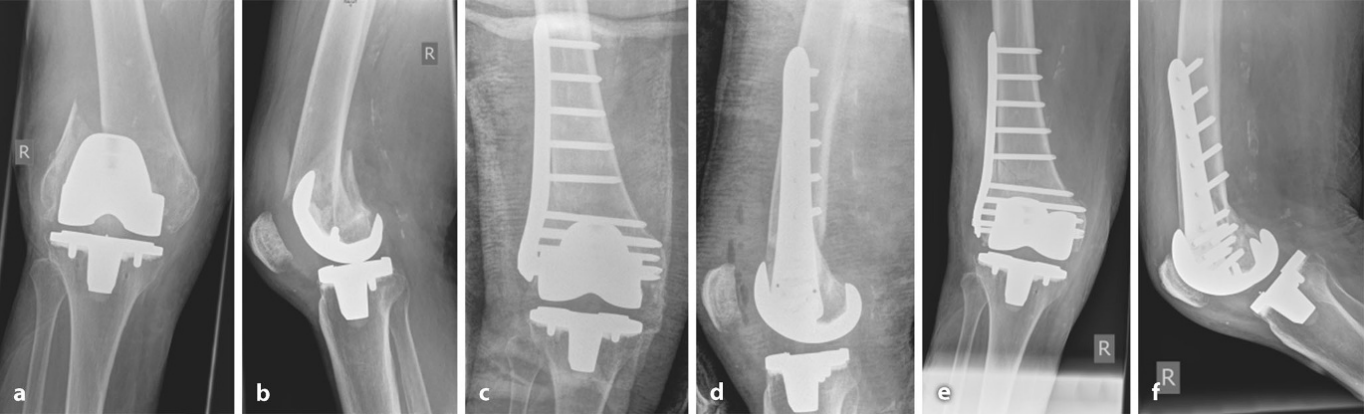

Die 83-jährige Patientin zog sich bei bekannter Osteoporose durch ein Niedrigenergierasanztrauma bei einem einfachen Sturz eine dislozierte periprothetische Femurfraktur rechts zu (Abb. 2).

Die operative Versorgung erfolgte mittels winkelstabiler Plattenosteosynthese innerhalb von 48 h nach Trauma bei einem ASA-Score von 4. Einteilung nach Rorabeck Typ 2.

Die 6‑Wochen-Röntgenkontrolle zeigte eine zunehmende knöcherne Konsolidierung ohne sekundäre Dislokation bei mäßiger Valgusabweichung.

## Diskussion

Ziele der unfallchirurgischen Therapie bei Patienten mit distaler periprothetischer Femurfraktur sind eine möglichst rasche Wiederherstellung der Mobilität und Funktion und eine Reduktion der Mortalität. Deshalb wurden in dieser retrospektiven Fallserie an einer möglichst homogenen Patientengruppe (periprothetische distale Femurfraktur, Versorgung mittels winkelstabiler Plattenosteosynthese) das klinische Outcome und die Mortalität mit Ihren Risikofaktoren nachuntersucht. Als mögliche Einflussparameter wurden Alter, Vorerkrankungen, Frakturmorphologie, Dauer bis zur operativen Versorgung, Operationstrauma, Komplikationen und stationärer Aufenthalt bis zur Rehabilitation untersucht.

Das Durchschnittsalter unseres Patientenkollektivs entspricht im Wesentlichen bereits der aktuellen Lebenserwartung in Deutschland nach Angabe des Statistischen Bundesamtes, und auch deshalb ist eine hohe Mortalität postoperativ zu erwarten [12]. Zudem sind viele Patienten in diesem Alter erheblich vorerkrankt, was sich in unserer Studie in mehr als zwei Drittel der Fälle mit einer ASA-Klassifikation von 3 oder 4 bzw. einem Charlson-Komorbiditätsindex von mehr als 2 Punkten bei 16 von 35 Patienten (im Gesamtkollektiv durchschnittlich 2,2 Punkte) widerspiegelt. Wie zu erwarten war, sind die Patienten mit mehr Vorerkrankungen tendenziell auch die älteren Patienten. Alle bisherigen Studien zu distalen Femurfrakturen, insbesondere periprothetischen distalen Femurfrakturen, berichten über vergleichbar hohe Mortalitätsraten.

Die Arbeitsgruppe um Myers et al. konnte bei distalen Femurfrakturen eine Einjahresmortalität von 13,4 % in einem Studienkollektiv von 283 Fällen (darunter 134 periprothetische Frakturen) aufzeigen [22]. Kammerlander et al. stellten bei einem Kollektiv von 43 Patienten mit distaler Femurfraktur (darunter 24 periprothetische Frakturen) eine Einjahresmortalität von 18,4 %, bzw. Dreijahresmortalität von 39,1 % fest [15]. Streubel et al. berichten über eine Einjahresmortalität von 27 % bei periprothetischen distalen Femurfrakturen und von 23 % für einfache distale Femurfrakturen [29]. Hoellwarth et al. untersuchten distale periprothetische Femurfrakturen und konnten für das plattenosteosynthetisch versorgte Kollektiv (*n* = 87) eine Dreimonats- bzw. Einjahresmortalität von 9 % bzw. 22 % erheben [11]. Unsere Ergebnisse aus Tab. 4 lassen sich in diese Reihe einfügen, mit tendenziell geringeren Mortalitätsraten.

Das klinische Outcome anhand des Lysholm-Scores in unserem Kollektiv zeigt eine hohe Heterogenität. Vergleichbare Daten finden sich in der Literatur nicht. In bisherigen Studien wurden allgemeine Informationen zur Selbstversorgung und zum Hilfsmittelgebrauch oder der Barthel-Index erfasst [11, 15, 16].

Wie Tab. 3 zu entnehmen ist, zeigt sich keine Korrelation zwischen der Frakturklassifikation nach Rorabeck mit dem Lysholm Score oder den Mortalitätsraten. Auch Streubel et al. [28] und Kim et al. [17] sehen vergleichbare funktionelle Ergebnisse zwischen sehr distalen Frakturen, die bis zur Prothesenkomponente reichen, und etwas weiter proximal gelegenen Frakturen [29]. Sie sehen deshalb die winkelstabile Plattenosteosynthese als adäquate Versorgung auch bei extrem distalen Frakturen mit schlechter Knochenqualität an. Auch in unserem Kollektiv zeigte die Versorgung mit winkelstabiler Plattenosteosynthese bei Grenzfällen mit radiologisch fraglicher Prothesenlockerung (Rorabeck 2–3) keine negativen Auswirkungen hinsichtlich von klinischem Outcome, Komplikationen und Mortalitätsrate. Präoperativ wurde die Prothese als nichtgelockert eingeschätzt, weshalb die operative Strategie der Plattenosteosynthese gewählt wurde. Eine sichere Prothesenlockerung hätte zwangsläufig ein anderes chirurgisches Vorgehen mit Revisionsprothese bedingt. Rein radiologisch ist aber aus unserer Sicht in Einzelfällen keine sichere Differenzierung möglich, weswegen hier eine Subgruppe mit den Typen 2 und 3 nach Rorabeck hinzugenommen wurde.

Bezüglich des Operationszeitpunktes werden in den bisherigen Studien unterschiedliche Ergebnisse berichtet. In mehreren Studien war eine positive Korrelation zwischen zeitlichem Abstand vom Trauma bis operativer Versorgung und der Mortalität ersichtlich. Bei einer zeitlichen Verzögerung um mehr als 4 Tage bei Patienten mit distaler Femurfraktur konnten Streubel et al. zeigen, dass sowohl die Sechsmonats- als auch Einjahresmortalität signifikant ansteigt [29]. Bei Hüftfrakturen bewirkt eine zeitliche Verzögerung um mehr als 2 Tage post Trauma einen Anstieg der Sterblichkeit [33]. Es ist daher naheliegend, dass sich auch bei distalen periprothetischen Frakturen eine Verzögerung um mehrere Tage nachteilig auf die Mortalität auswirkt. Trotzdem finden sich in der Literatur auch Ergebnisse, die wie in unserem Kollektiv keinen signifikanten Unterschied hinsichtlich der Mortalität bei Patienten mit Versorgung innerhalb und nach 48 h sehen [11]. Trotz dieser Ergebnisse halten wir eine möglichst frühzeitige operative Versorgung, soweit möglich, für sinnvoll. Als weiterer möglicher Prädiktor für das klinische Outcome wurde die Invasivität des operativen Eingriffs untersucht. Operationsdauer und intraoperativer Blutverlust waren jedoch ohne erkennbaren Einfluss auf das Langzeitergebnis.

Gemessen an der Komplexität der Verletzung und den Vorerkrankungen der Patienten zeigte sich eine moderate Rate an Komplikationen (16,7 % Pseudarthrose, 25 % Komplikationen gesamt). Ähnliche Werte zur Pseudarthrosenrate werden von Ebrahim et al. mit 10–20 % angegeben [5] und in einer prospektiven Studie von Eschbach et al. mit 37 plattenosteosynthetisch versorgten Rorabeck-1- und Rorabeck-2-Frakturen mit 22 % Komplikationen mit operativer Revision [6].

Die Vorerkrankungen der Patienten, die mit der ASA-Klassifikation und dem Charlson-Komorbiditätsindex erfasst wurden, scheinen einen negativen Einfluss auf den Lysholm-Score zu haben. Eine Signifikanz wurde aufgrund der Fallzahl und Variabilität des Parameters in unserem Kollektiv nicht erreicht (Tab. 4). Ein signifikanter Zusammenhang besteht zwischen Mortalität und ASA-Score/Charlson-Index und zwischen Mortalität und Patientenalter. Der präoperative Allgemeinzustand des Patienten stellt den wichtigsten Einflussfaktor auf das klinische Outcome und die Mortalität in unserem Patientenkollektiv dar, wesentlicher als beispielsweise die Frakturklassifikation, präoperative Liegedauer, stationäre Aufenthaltsdauer oder intraoperative Parameter.

Zu einem ähnlichen Ergebnis kommt die bisher größte vergleichende retrospektive Studie von Hoellwarth et al. mit 140 eingeschlossenen Patienten. Weder die Frakturform noch die Versorgungsstrategie (Plattenosteosynthese *n* = 87 vs. Prothesenwechsel *n* = 53) haben laut den Autoren einen signifikanten Einfluss auf das klinische Outcome (Selbstständigkeit und Hilfsmittel) oder die Mortalität nach 3 Monaten und einem Jahr [11]. Lediglich das Alter und die Komorbiditäten zeigen einen signifikant negativen Einfluss auf die Einjahresmortalität in beiden Subgruppen. Die beiden Versorgungsstrategien weisen zudem interessanterweise keine Unterschiede hinsichtlich der weiteren Mobilisierung mit Vollbelastung und Reoperationsraten auf.

Bislang wurde der Einfluss der bereits liegenden Knieprothese auf das Operationsverfahren kaum untersucht. Hier wurde kürzlich von der Universitätsklinik Leipzig eine umfassendere Einteilung der distalen periprothetischen Frakturen vorgestellt [7]. Die Forderung nach einer weiteren spezifizierenden Einteilung ist verständlich, da die Rorabeck-Einteilung lediglich 2 Kriterien, nämlich Dislokation und Prothesenlockerung, berücksichtigt und der Komplexität der Verletzungen oftmals nicht gerecht wird. Durch die neue Klassifikation wird eine genauere Abgrenzbarkeit ermöglicht und die Art der liegenden Prothese mitberücksichtigt. In Anbetracht der Heterogenität des Patientenkollektivs und der Frakturmorphologien erscheint dies notwendig. Da sich diese Einteilung noch nicht im täglichen klinischen Alltag wiederfindet, wurde in der vorliegenden Untersuchung noch die Rorabeck-Klassifikation verwendet. Das Problem einer radiologisch fraglich gelockerten Prothese scheint aber aus unserer Sicht weiter ungelöst, und die bisher verfügbaren Daten deuten darauf hin, dass im Zweifel auch der Versuch einer Plattenosteosynthese gerechtfertigt ist.

Ob durch optimierte Schmerztherapie und postoperative Mobilisierung die Mortalität in diesem hochbetagten Patientenkollektiv noch verbessert werden kann, bleibt Gegenstand weiterer Untersuchungen, zumal gezeigt werden konnte, dass geriatrische Patienten oftmals eine Teilbelastung ohnehin schwerlich oder kaum umsetzen können [14]. Wir sind daher mittlerweile dazu übergegangen, die Patienten zugunsten einer zügigeren Mobilisierung postoperativ schmerzabhängig vollbelasten zu lassen – entgegen den Bedingungen der vorliegenden Studie. Der Ausbau intensiver und interdisziplinärer geriatrischer Behandlungskonzepte für dieses Patientenkollektiv ist ein sicher wichtiger und richtiger Schritt [8, 16, 25].

### Limitationen

Die große Variabilität der Sterberaten zwischen den berichteten Studien kann in der unterschiedlichen länderspezifischen Lebenserwartung, den unterschiedlichen Komorbiditäten bei kleinen Patientenkollektiven und den verschiedenen Krankenhausressourcen begründet sein [21]. Ebenso beeinflussen länderspezifische Unterschiede in der weiteren Behandlung mit intensiver zeitnaher Rehabilitationsphase oder fehlender weiterer Versorgung das Outcome der Patienten.

Das klinische Outcome ist im geriatrischen Kollektiv schwierig zu evaluieren, quantitativ aufgrund der geringen Quote an Patientenwiedervorstellungen und qualitativ ist die Anamnese bei geriatrischen Patienten bezüglich Selbstständigkeit und Gelenkfunktion deutlich erschwert. Deshalb konnte auch die postoperative Nachbehandlung nicht in diese Untersuchung einbezogen werden, wobei eine fehlende Mobilisierung ein erheblicher Einflussfaktor auf die Mortalität sein kann.

Die geringe Fallzahl ist eine wesentliche Limitation der Studie, wobei die meisten Studienkollektive zu dieser Thematik ähnliche Fallzahlen aufweisen. Detaillierte Informationen zum weiteren Verlauf bei Komplikationen sind leider nicht für alle Patienten vorhanden.

Für weitere Untersuchungen bezüglich dieser Frakturentität wären multizentrische prospektive Studien, inklusive einer detaillierten Betrachtung der präoperativen und postoperativen Situation, wünschenswert.

## Fazit für die Praxis

Zusammenfassend lässt sich festhalten, dass die distale periprothetische Femurfraktur unter allen Frakturen eine seltene, aber komplexe und schwerwiegende Verletzung darstellt, die vornehmlich geriatrische multimorbide Patienten erleiden. Die Mortalität im kurzen und im mittelfristigen Verlauf ist hoch. In unserem Kollektiv sind Vorerkrankungen der wesentliche Risikofaktor für die Ein- und Dreijahresmortalität und beeinflussen die klinische Funktion. Die Frakturmorphologie zeigte keinen Einfluss auf diese Parameter. Auch Grenzfälle mit radiologisch fraglicher Prothesenlockerung konnten in diesem Kollektiv suffizient mittels winkelstabiler Plattenosteosynthese versorgt werden.
